# Supplementary material for: Four ribbons of double-layer graphene suspending masses for NEMS applications
Source: Microsyst Nanoeng. 2024 Oct 21;10:150. doi: 10.1038/s41378-024-00799-x (PMC11493961; doi:10.1038/s41378-024-00799-x)
Supplement: Supplementary file 1 — Supporting information [file 41378_2024_799_MOESM1_ESM.docx]

**Supporting information for**

**Four ribbons of double-layer graphene suspending masses for NEMS applications**

Xuge Fan^1^^,2,3^*, Chang He^1^, Jie Ding^2^*, Sayedeh Shirin Afyouni Akbari^4^ and Wendong Zhang^5,6^*

^1^Advanced Research Institute of Multidisciplinary Sciences, Beijing Institute of Technology, 100081 Beijing, China.

^2^School of Integrated Circuits and Electronics, Beijing Institute of Technology, 100081 Beijing, China.

^3^Center for Interdisciplinary Science of Optical Quantum and NEMS Integration, Beijing 100081, China.

^4^Advanced NEMS Group, École Polytechnique Fédérale de Lausanne (EPFL), 1015 Lausanne, Switzerland.

^5^State Key Laboratory of Dynamic Measurement Technology, North University of China, Taiyuan 030051, China.

^6^National Key Laboratory for Electronic Measurement Technology, School of Instrument and Electronics, North University of China, Taiyuan 030051, China.

*Email: xgfan@bit.edu.cn, jie.ding@bit.edu.cn, wdzhang@nuc.edu.cn

**CONTENTS**

**Table S1.** Types and dimensions of all measured devices

**Figure S1.** Dynamic mechanical characterization of two-ribbon devices by measuring the amplitude of thermomechanical noise in air using LDV

**Figure S2.** Comparison of built-in stresses of devices 1-17 that were simulated at different Young’s moduli

**Text S1.** Analytical model for extraction of Young’s modulus

**Table S1. Types and dimensions of all measured devices.**

| **Device**  **type** | **Device number** | **Trench width** | **Mass size** | **Single ribbon width** | **Single ribbon length** | **Notes** |
| --- | --- | --- | --- | --- | --- | --- |
| **Two- ribbon devices** | Device 1 | 2 µm | 15 µm × 15 µm × 16.4 µm | ~5 µm | 2 µm |  |
|  | Device 2 | 2 µm | 20 µm × 20 µm × 16.4 µm | ~5 µm | 2 µm |  |
|  | Device 3 | 2 µm | 25 µm × 25 µm × 16.4 µm | ~5 µm | 2 µm |  |
|  | Device 4 | 2 µm | 30 µm × 30 µm × 16.4 µm | ~5 µm | 2 µm |  |
| **Four- ribbon-cross devices** | Device 5 | 2 µm | 10 µm × 10 µm × 16.4 µm | ~5 µm | 2 µm | One ribbon was defective |
|  | Device 6 | 2 µm | 15 µm × 15 µm × 16.4 µm | ~5 µm | 2 µm |  |
|  | Device 7 | 2 µm | 20 µm × 20 µm × 16.4 µm | ~5 µm | 2 µm |  |
|  | Device 8 | 2 µm | 25 µm × 25 µm × 16.4 µm | ~5 µm | 2 µm |  |
|  | Device 9 | 2 µm | 30 µm × 30 µm × 16.4 µm | ~5 µm | 2 µm |  |
|  | Device 10 | 2 µm | 40 µm × 40 µm × 16.4 µm | ~5 µm | 2 µm |  |
|  | Device 11 | 2 µm | 50 µm × 50 µm × 16.4 µm | ~5 µm | 2 µm |  |
| **Four- ribbon-parallel devices** | Device 12 | 2 µm | 15 µm × 15 µm × 16.4 µm | ~5 µm | 2 µm | One ribbon was defective |
|  | Device 13 | 2 µm | 20 µm × 20 µm × 16.4 µm | ~5 µm | 2 µm |  |
|  | Device 14 | 2 µm | 25 µm × 25 µm × 16.4 µm | ~5 µm | 2 µm |  |
|  | Device 15 | 2 µm | 30 µm × 30 µm × 16.4 µm | ~5 µm | 2 µm |  |
|  | Device 16 | 2 µm | 40 µm × 40 µm × 16.4 µm | ~5 µm | 2 µm |  |
| **Four- ribbon-cross devices** | Device 17 | 4 µm | 20 µm × 20 µm × 16.4 µm | ~5 µm | 4 µm | One ribbon was defective |

**
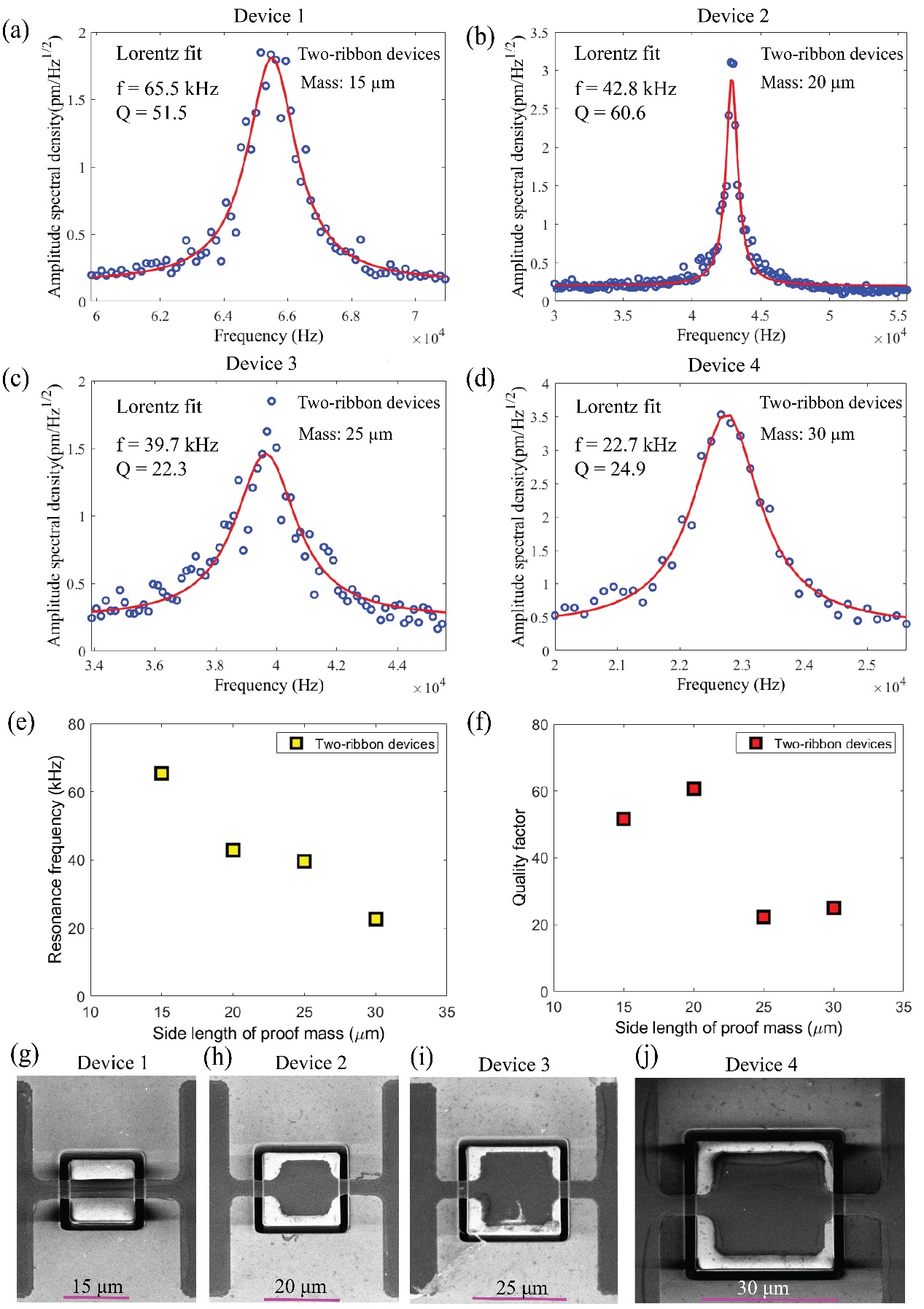
**

**Figure S1. Dynamic mechanical characterization of two-ribbon devices by measuring the amplitude of thermomechanical noise in air using LDV.** (a-d) Thermomechanical noise peak of devices 1-4 using LDV, where the red solid lines in (a-d) were based on Lorentz fitting and extracted resonance frequencies and quality factors. The four two-ribbon devices have identical trench width (2 µm) and ribbon width (5 µm) but different proof mass dimensions (15 µm × 15 µm × 16.4 µm in (a); 20 µm × 20 µm × 16.4 µm in (b); 25 µm × 25 µm × 16.4 µm in (c) and 30 µm × 30 µm × 16.4 µm in (d)). (e) Resonance frequencies of devices 1-4 versus the side length of proof mass of devices 1-4. (f) Quality factor of devices 1-4 versus the side length of the proof mass of devices 1-4. (g-j) SEM images of devices 1-4 in (a-d), respectively.

**
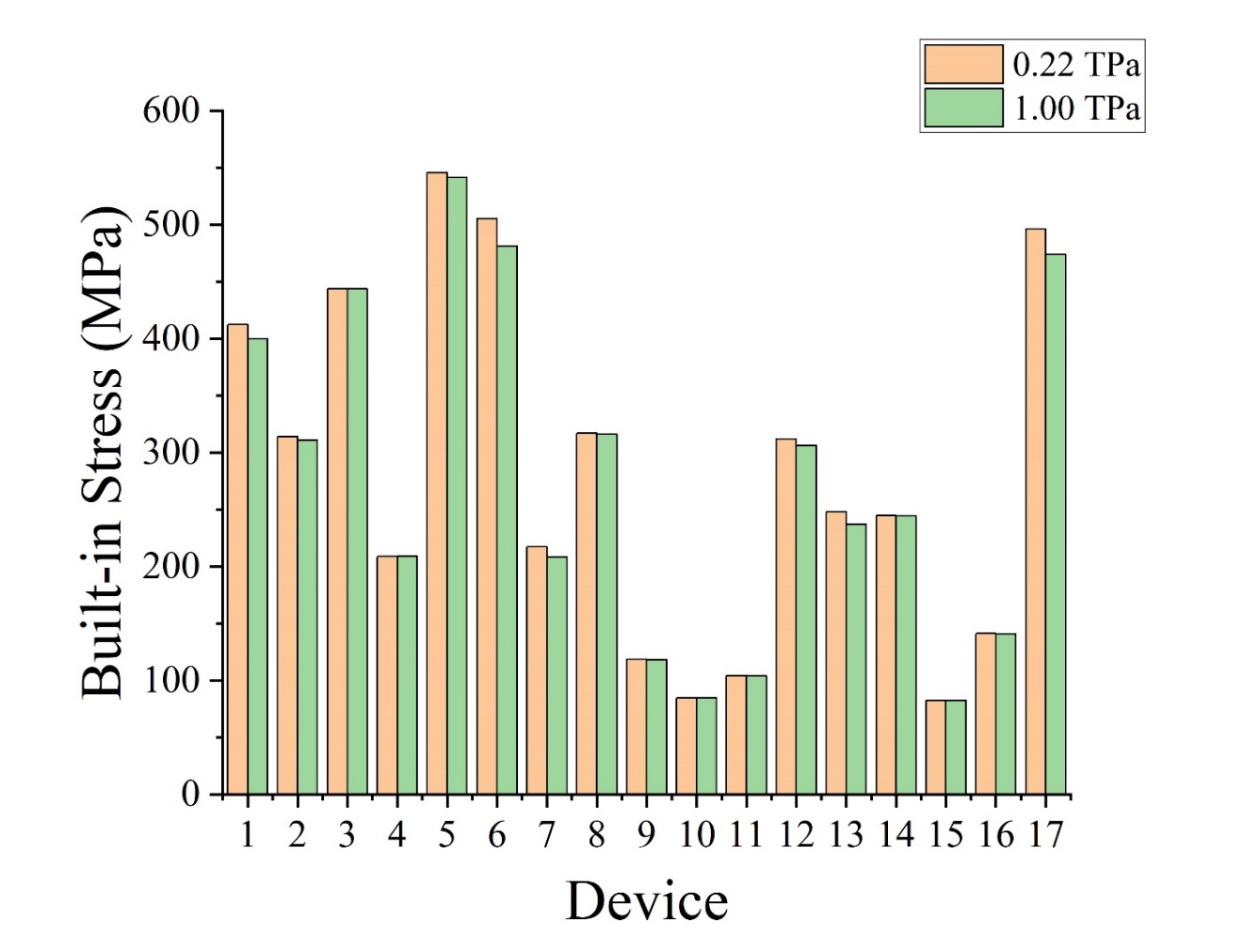
**

**Figure S2.** **Built-in stresses of devices 1-17 that were obtained by FEA simulation at different Young’s moduli (0.2 TPa and 1 TPa) of double-layer graphene.** The results show that the magnitude of the Young’s modulus of double-layer graphene has ignorable impact on the extraction of the built-in stress of devices 1-17.

**Text S1**

For large ribbon displacements compared to the ribbon thickness, the displacement at the centre of the ribbon induced by a centre-point load can be described by^1^

$F=\left( \frac{16EWH^{3}}{L^{3}}+\frac{4\sigma_{0}WH}{L} \right)Z+ \frac{8EWH}{L^{3}}Z^{3}$ (s1)

where F is the load applied at the centre of the ribbon, Z the resulting deflection of the ribbon at its centre, E the Young’s modulus, W the ribbon width, H the ribbon thickness, L the total ribbon length, and $\sigma_{0}$ the average residual built-in stress in the ribbon. From equation s1, the linear spring constant can be obtained by

$K=\frac{16EWH^{3}}{L^{3}}+\frac{4\sigma_{0}WH}{L}$ (s2)

According to equations 1 and s2, the average built-in stress can be obtained by

$\sigma_{0}= \frac{L}{4WH}(m\left( 2\pi f \right)^{2}-\frac{16EWH^{3}}{L^{3}})$ (s3)

By using equation s3, the built-in stress in the graphene ribbons of the two-ribbon devices in **Figure S1** can be estimated. When using a Young’s modulus value of E = 0.22 TPa for the double-layer graphene (previously extracted for comparable double-layer graphene devices^1^), the built-in stresses of the two-ribbon devices (devices 1-4) in **Figure S1** were estimated to be 435.78 MPa, 331.24 MPa, 444.88 MPa and 210.28 MPa, respectively, all of which are of the same order of magnitude as those we extracted previously for similar devices.^1^ It should be noted that the extraction of built-in stress does not depend on the magnitudes of the Young’s modulus. For instance, if we use the Young’s modulus of 1 TPa for the double-layer graphene, the built-in stresses of two-ribbon devices (devices 1-4) were 435.69 MPa, 331.15 MPa, 444.79 MPa and 210.19 MPa, respectively, all of which were almost equal to the corresponding values based on the use of Young’s modulus of 0.22 TPa, with only about 0.02% variance.

**References**

1. Fan, X. *et al.* Graphene ribbons with suspended masses as transducers in ultra-small nanoelectromechanical accelerometers. *Nat. Electron.* **2**, 394–404 (2019).
